# Antihypertensive Potential of Combined Extracts of Olive Leaf, Green Coffee Bean and Beetroot: A Randomized, Double-Blind, Placebo-Controlled Crossover Trial

**DOI:** 10.3390/nu6114881

**Published:** 2014-11-05

**Authors:** Rachel H.X. Wong, Manohar L. Garg, Lisa G. Wood, Peter R.C. Howe

**Affiliations:** Clinical Nutrition Research Centre and Nutraceuticals Research Group, School of Biomedical Sciences and Pharmacy, Faculty of Health and Medicine, University of Newcastle, Callaghan, NSW 2308, Australia; E-Mails: rachel.wong@newcastle.edu.au (R.H.X.W.); manohar.garg@newcastle.edu.au (M.L.G.); lisa.wood@newcastle.edu.au (L.G.W.)

**Keywords:** ambulatory blood pressure, olive leaf extract, chlorogenic acid, hypertension, cholesterol, insulin sensitivity

## Abstract

Extracts of olive leaf, green coffee bean and beetroot may deliver cardiovascular benefits. This study sought to evaluate the effects of regularly consuming a combination of these extracts on blood pressure (BP), arterial compliance, blood lipids, blood glucose and insulin sensitivity. A double-blind randomised placebo-controlled crossover trial was conducted in adults with untreated high normal or borderline elevated BP. They were randomised to take an active supplement, comprising 500 mg olive leaf extract, 100 mg green coffee bean extract and 150 mg beet powder, or a matching placebo twice daily for six weeks, followed by the alternate supplement for a further six weeks. Assessments of 24-h ambulatory BP (ABP), clinic BP arterial compliance (pulse-wave analysis), blood lipids, blood glucose and insulin were obtained at baseline and at the end of each treatment phase. Baseline clinic BP in 37 overweight middle-aged men and women who completed the trial averaged 145/84 mmHg. There was no significant effect of treatment on ABP or any other outcome measure. The failure to confirm prior evidence of the antihypertensive benefits of these extracts emphasises the importance of placebo control and the value of ABP monitoring. Further dose-response evaluation of olive leaf, green coffee bean or beetroot extracts is required to confirm or refute the purported benefits.

## 1. Introduction

Mediterranean societies have used olive tree leaves (*Olea europaea* L.) in traditional medicine. The olive leaf is believed to have antihypertensive, hypocholesterolemic, hypoglycaemic and anti-inflammatory properties, which may be mediated by oleuropein, the predominant bioactive compound in olive leaf, and/or by other polyphenolic constituents [1,2,3,4,5]. Susalit *et al*. [3] reported reductions in systolic (SBP) and diastolic (DBP) pressure of 11.5/4.8 mmHg in untreated hypertensive adults after taking an olive leaf extract (1,000 mg/d) for eight weeks. A similar magnitude of BP reduction was also reported in a twins study after consuming the same dose of olive leaf extract daily for six weeks [2]. Each study also reported reductions of LDL cholesterol. However, these studies lacked placebo comparisons; thus, further randomised placebo-controlled trials are needed to confirm the potential cardiometabolic health benefits of olive leaf extracts in humans.

The constituents of olive leaf responsible for any antihypertensive or cholesterol lowering effects are yet to be fully identified; however, a wide variety plant polyphenols are known to have antihypertensive potential as a result of their ability to enhance arterial dilatation, primarily via increased nitric oxide (NO) production in endothelial cells. Sources of these bioactives include decaffeinated green coffee bean extract and beetroot, as well as olive leaf. Chlorogenic acid, the major vasoactive ingredient in green coffee bean extract, has been shown to have a dose-dependent antihypertensive effect (up to 4.7/3.2 mmHg over 28 days) in untreated mildly hypertensive men; the reduction was significant at 93 mg/day (50 mg of chlorogenic acid) [6]. Chlorogenic acid appears to increase NO bioavailability, which could account for its BP lowering effects [7]. Likewise, beetroot juice has been recently acknowledged as an excellent source of nitrate, which can be retroconverted to NO, with the potential to enhance vasodilatation; acute reduction of BP has been reported with as little as 100 g of beetroot juice [8,9].

Clinic BP recordings, as used in the abovementioned studies, are highly variable, subject to artefacts (e.g., white coat hypertension) [10] and undergo regression to the mean with repeated measurement. All of these artefacts can be minimised through the use of 24-h ambulatory BP monitoring (ABPM), together with average daytime and nocturnal ABPM [10]. This gold standard approach to the assessment of BP change is yet to be applied in clinical trials evaluating the antihypertensive efficacy of olive leaf, green coffee bean or beetroot extracts, either alone or in combination.

The present study sought to confirm the antihypertensive efficacy of the olive leaf extract in a placebo controlled comparison using 24-h ABPM in adults with mildly elevated, but untreated BP. In addition, it aimed to evaluate the effect of regular twice daily consumption of a novel formulation, which combined 500 mg of the olive leaf extract, 100 mg of green coffee bean extract (equivalent to 46.5 mg of chlorogenic acid) and 150 mg of beet powder in a single tablet on 24-h, daytime and nocturnal ABP. We hypothesised that this combination of extracts would have additive or synergistic effects on BP, LDL cholesterol glucose and insulin sensitivity. Evaluating the combined efficacy of dietary supplements that are available individually has potential implications for consumers desirous of obtaining a more potent formulation [11]. The results of this study were intended to inform the development of a more potent supplement for BP management.

## 2. Experimental Section

### 2.1. Study Design

A 12-week randomised, double-blind, placebo-controlled cross-over human dietary intervention trial with the combined formulation (as described above) or matching placebo (of identical appearance) was conducted at the Hunter Medical Research Institute, Clinical Nutrition Research Centre, University of Newcastle, in accordance with the principles of International Conference on Harmonisation’s Good Clinical Practice. The study was approved by the Human Research Ethics Committee of the University of Newcastle (H-2013-0131; approved on July 26, 2013), registered with the Australia and New Zealand Clinical Trials Registry (ACTRN12613000841774) and the Therapeutic Goods Administration of Australia.

### 2.2. Study Population

Volunteers residing in the Hunter region of New South Wales, Australia, were recruited from the general public via the Hunter Medical Research Institute research volunteer registry and public media announcements. All volunteers provided written informed consent prior to commencing the trial.

Inclusion criteria were age 18–80 years, body mass index (BMI) between 20 and 35 kg/m^2^ (inclusive) and BP recorded at baseline/screening Visit 1 between 130–160 mmHg systolic and 85–100 mmHg diastolic. Exclusion criteria were as follows: (1) smokers or taking nicotine therapy; (2) taking antihypertensive medication or insulin; (3) pregnant or currently breastfeeding; (4) unwilling to wear the ABP monitor or undergo BP monitoring for 24 h; (5) unwilling to maintain habitual diet and physical activity during the intervention; (6) currently consuming dietary supplements containing extracts of olive leaf, green coffee bean or beet.

### 2.3. Assessment Protocol

**Figure 1:**
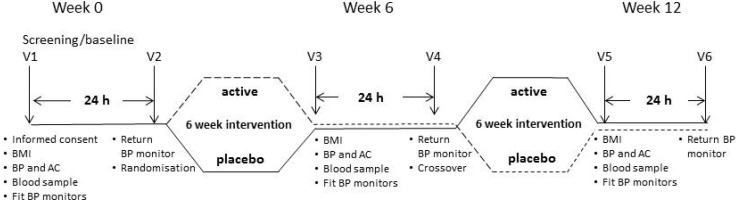
Outline of study design. During baseline/screening Visit 1 (Week 0), participants underwent assessments of body mass index (BMI), clinic blood pressure (BP), arterial compliance (AC), 24-h ambulatory BP (BP monitors), fasting blood lipids, glucose and insulin levels to confirm suitability before randomisation to treatment. These assessments were repeated at Visit 3 (Week 6), before participants switched over to the alternate treatment (Visit 4, Week 6) and again at Visit 5 (Week 12).

Enrolled participants were required to visit HMRI on 6 occasions over 12 weeks, as shown in Figure 1. They attended the clinic at Visits 1, 3 and 5 following an overnight fast of 8 h. Volunteers were screened for eligibility at baseline, including assessment of clinic BP, arterial compliance (AC), 24-h ABP, fasting blood lipids, glucose and insulin, before being randomised to active treatment or a matching placebo. After returning their ABP monitors, they received the supplements to which they had been randomised and were instructed to maintain their habitual diet and physical activity throughout the intervention. Allocation to either treatment phase was based on randomisation by the minimisation method (age, gender and BMI) to ensure well-balanced groups in the first intervention phase [12]. Outcome measures were obtained after Week 6 and Week 12 at Visits 3 and 5. At Visit 4 in Week 6, following the measurement of 24-h ABPM, participants crossed over to the other treatment.

### 2.4. Supplement and Treatment Regime

The combined formulation and placebo were delivered in tablets of identical appearance. The active tablet was comprised of 500 mg of olive leaf extract (Benolea^®^ EFLA^®^942, Frutarom Ltd.; comprises 16%–24% oleuropein and ≥30% other polyphenols), 100 mg of green coffee bean extract (Svetol™, Naturex; comprised of 45%–50% chlorogenic acid, 10%–15% 5-caffeoylquinic acid, 50%–55% other polyphenols and 0%–2% caffeine) and 150 mg of Sensient^®^ No.03600 beetroot powder per tablet. The placebo tablets comprised inert excipients (microcrystalline cellulose, stearic acid, cellulose powder, croscarmellose sodium and silicon dioxide).

Participants were instructed to take 2 tablets daily, preferably at the same time with meals, and to record each intake in the assigned supplement diary. If a dose was missed, participants were requested to indicate the reason for missing and not carrying over the dose to the next day. Any changes in medication and/or dietary supplement intake during the intervention were to be self-reported in the supplement diary. At the end of 6 weeks, the bottle, diary and any unused tablets were returned. At the end of the trial, all tablets were manually counted and tallied with the corresponding diary records to monitor compliance.

### 2.5. Blinding

The olive leaf formulation and placebo tablets were identical in appearance, and each was dispensed in sealed white opaque containers, which were identifiable only by code numbers. An independent investigator randomised participants to treatments and assigned container code numbers. The investigational product was safely stored in a locked and limited access area. Trial investigators remained blinded until all data analysis had been performed.

### 2.6. Outcome Assessments

#### 2.6.1. Resting Clinic Blood Pressure and Arterial Compliance

Standing height and body weight were measured before vascular assessments commenced. Seated BP, heart rate (HR) and AC assessments were performed after resting in a seated position for 10 min. Four consecutive readings were taken at 5-min intervals by automated oscillometry using a standard BP cuff over the left brachial artery (to assess BP and HR) and a tonometer (for pulse wave analysis of AC) positioned perpendicularly over the right radial artery with a sensitivity of at least 18% (to assess large and small artery elasticity indexes) using a Cardiovascular Profiler (HDI Cardiovascular Profiler CR2000 Minnesota, USA). BP measurements were performed by a single investigator, in accordance with the guidelines [13]. Discarding the first reading, an average of the remaining measurements was recorded for analysis. If average BP was greater than 160/100 mmHg at the baseline/screening Visit 1 (Week 0), the participant would not be included in the trial and referred to their health professional for further evaluation.

#### 2.6.2. 24-h Ambulatory Blood Pressure Monitoring

An appropriately-sized BP cuff was placed firmly around the upper non-dominant arm, centred over the brachial artery, with the ABP monitor (TM-2430, A & D Medical, Australia) worn on a waist strap. ABP monitors were programmed to record at 15-min intervals during the day (07.00–22.00 h) and at 30-min intervals at night (22.00–07.00 h). The BP readings were programmed not to display to minimise the influence of the knowledge of the participant. The cuff and monitor were removed briefly for bathing or water-based activities and remained in place at all other times during the 24-h recording period. Each participant was assigned the same monitor and cuff size on each occasion. Participants also kept an activity diary to enable BP values to be related to their activities. The 24-h ABP data were downloaded and analysed using Doctor Pro 3 for Windows™ (TM-2430; version 3.03.00, A & D Medical, Australia).

#### 2.6.3. Biochemistry

Fasting blood samples were collected by a certified phlebotomist at the Hunter Area Pathology Services, John Hunter Hospital, New South Wales, Australia, and sent to the Hunter Area Pathology Services laboratory for analyses of fasting serum lipids, glucose and insulin levels. The Homeostasis Model Assessment of Insulin Resistance (HOMA-IR) Index was determined by dividing the product of glucose and insulin levels by 22.5 [14].

### 2.7. Sample Size and Statistical Analysis

This study was powered to detect differences between the combined formulation and placebo treatment. Assuming no order effect with the 2-way randomisation and no carry-over effect, a total of 34 completers would give 80% power to detect a significant (*p* < 0.05) difference in average 24-h ABP of at least 3/2 mmHg (SBP/DBP). Forty participants were recruited to allow for attrition.

All statistical analyses were performed on IBM^®^ SPSS^®^ Statistics Version 21.0 (Chicago, Illinois, USA). Data were checked for: (1) an order effect with the crossover design by comparing the treatment difference (*i.e.*, active, placebo) between those randomised initially to active treatment (Group 1) and those who received the placebo (Group 2); and (2) a difference in the measures of Group 1 and the group at baseline using *t*-tests (data not shown). In the intention-to-treat analysis, the effect of treatment on the primary outcome was the mean within-subject difference in ABPM between the active and placebo treatments, assessed from averaged 24-h daytime and nocturnal measurements of SBP, DBP, HR and mean arterial pressure (MAP) at the end of each 6-week intervention phase. This was determined using paired *t*-tests. Secondary outcomes (clinic BP, HR and AC, fasting serum lipids, glucose, insulin and HOMA-IR) were similarly tested; however, a modified Bonferroni adjustment was made to the levels of significance for multiple comparisons.

There were sufficient numbers in each treatment arm to additionally perform a parallel comparison; independent *t*-tests were used to compare pre-post treatment differences in the active and placebo arms during the first intervention phase. In addition, we determined whether there were any treatment differences between sexes, as there was a good gender split.

**Figure 2:**
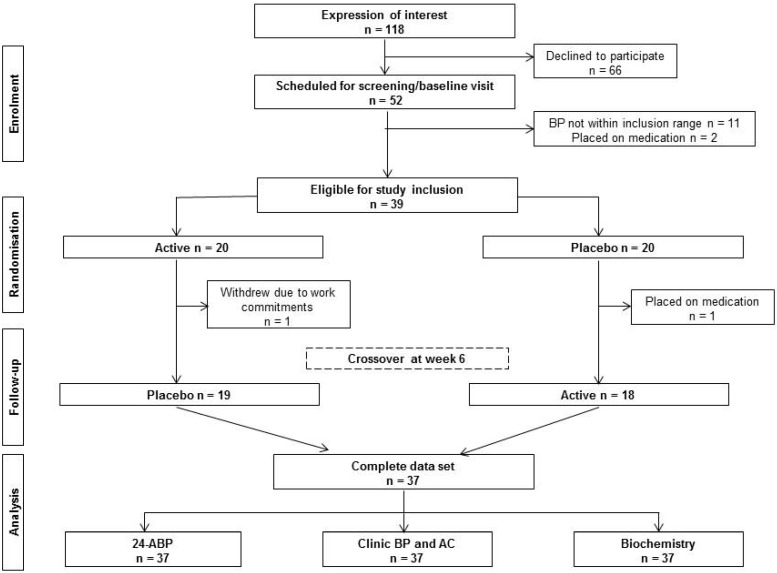
Consolidated Standards of Reporting Trials (CONSORT) diagram.

## 3. Results

### 3.1. Participant Disposition

The study was conducted between September and December, 2013. Figure 2 shows the flow of participants from recruitment to data analysis. Of the 52 participants who were invited to a screening/baseline visit, 13 participants failed to meet the inclusion criteria for the study. Two of these had been placed on antihypertensive medication before their screening/baseline visit. The remaining 11 excluded participants failed to meet the inclusion criterion for clinic BP. Of the 39 participants who were enrolled, 37 completed the 12-week intervention. The two withdrawals occurred prior to the first assessment point in Week 6. One participant commenced antihypertensive medication, and one participant withdrew due to work commitments. Consequently, 37 participants completed all assessment time points and were included in the primary and secondary outcomes analyses. Participant compliance with the intervention was greater than 97% for both the combined formulation arm (98.5% ± 0.05%) and placebo arm (97.6% ± 0.05%); therefore, all 37 datasets were included for analysis.

Baseline characteristics of the participants are shown in Table 1, Table 2 and Table 3. Participants were slightly overweight middle-aged adults with mildly-elevated clinic BP and 24-h ABP. With the exception of total cholesterol, their glucose, insulin, triglycerides, HDL and LDL cholesterol were within the acceptable ranges set forth by the National Heart Foundation of Australia and the Cardiac Society of Australia and New Zealand [15,16]. There were no differences in baseline characteristics between those randomised initially to active or placebo treatments.

**Table 1:** Participant characteristics, clinic BP (blood pressure), HR (heart rate) and AC (arterial compliance) at baseline (Week 0) and at the end of the active phase and placebo phase (*n* = 37). SBP, systolic BP; DBP, diastolic BP.

	Baseline	End of Active Phase		End of Placebo Phase		Difference (Active, Placebo)		
	Mean ± SD	Mean ± SEM		Mean ± SEM		Mean ± SEM		*p*
Gender (male/female)	20/17							
Age (years)	58.5 ± 10.7							
BMI (kg/m^2^)	27.2 ± 3.7	27.3 ± 0.6		27.3 ± 0.6		0.05 ± 0.06		0.382
Clinic SBP (mmHg)	145.4 ± 7.8	135.3 ± 1.6		133.9 ± 1.5		1.41 ± 1.44		0.332
Clinic DBP (mmHg)	84.4 ± 6.5	79.1 ± 1.2		80.0 ± 1.4		−0.94 ± 0.88		0.293
Clinic HR (bpm)	66.6 ± 8.4	64.8 ± 1.5		64.0 ± 1.5		0.87 ± −0.58		0.233
Clinic large artery elasticity index (mL/mmHg × 10)	11.5 ± 4.1	12.8 ± 0.6		14.2 ± 0.7		−1.40 ± 0.56		0.017
Clinic small artery elasticity index (mL/mmHg × 100)	3.8 ± 2.1	4.2 ± 0.3		3.9 ± 0.3		0.28 ± 0.25		0.277

**Table 2:** Average 24 h, daytime and nocturnal ABP at baseline and the end of each phase (*n* = 37).

	Baseline	End of Active Phase	End of Placebo Phase	Difference (Active, Placebo)	
	Mean ± SD	Mean ± SEM	Mean ± SEM	Mean ± SEM	*p*
24-h ambulatory BP average					
SBP (mmHg)	135.8 ± 9.8	134.6 ± 1.8	132.8 ± 1.5	1.78 ± 1.18	0.138
DBP (mmHg)	78.0 ± 6.3	79.7 ± 1.0	78.7 ± 0.9	1.05 ± 0.67	0.122
MAP (mmHg)	98.2 ± 7.0	97.7 ± 1.2	96.3 ± 1.0	1.32 ± 0.79	0.102
HR (bpm)	72.7 ± 8.2	72.4 ± 1.3	72.2 ± 1.3	0.24 ± 0.73	0.744
Daytime BP average (07.00–22.00 h)					
SBP (mmHg)	140.6 ± 9.7	139.4 ± 1.8	137.2 ± 1.4	2.11 ± 1.33	0.123
DBP (mmHg)	83.0 ± 6.5	82.8 ± 1.0	81.7 ± 1.0	1.16 ± 0.77	0.141
MAP (mmHg)	101.9 ± 7.10	101.4 ± 1.1	99.8 ± 1.0	1.60 ± 0.86	0.071
HR (bpm)	75.4 ± 8.3	75.2 ± 1.4	74.9 ± 1.4	0.30 ± 0.84	0.724
Nocturnal BP average (22.00–07.00 h)					
SBP (mmHg)	120.0 ± 11.7	119.6 ± 2.5	118.9 ± 2.1	0.68 ± 1.37	0.624
DBP (mmHg)	70.2 ± 7.3	70.0 ± 1.4	69.2 ± 1.3	0.76 ± 0.84	0.375
MAP (mmHg)	86.4 ± 8.4	86.2 ± 1.7	85.5 ± 1.4	0.73 ± 0.96	0.452
HR (bpm)	64.2 ± 9.0	64.0 ± 1.4	64.3 ± 1.2	−0.32 ± 0.76	0.670

**Table 3:** Blood biochemistry: total, HDL and LDL cholesterol, triglyceride, glucose and insulin and HOMA-index of insulin resistance at baseline and the end of each phase (*n* = 37).

	Baseline	End of Active Phase	End of Placebo Phase	Difference (Active, Placebo)	
Biochemistry	Mean ± SD	Mean ± SEM	Mean ± SEM	Mean ± SEM	*p*
Glucose (mmol/L)	5.18 ± 0.64	5.20 ± 0.11	5.15 ± 0.12	0.05 ± 0.07	0.440
Insulin (mmol/L)	6.31 ± 2.90	7.32 ± 0.52	6.80 ± 0.58	0.51 ± 0.37	0.177
HOMA-IR	1.40 ± 0.69	1.72 ± 0.14	1.58 ± 0.15	0.14 ± 0.10	0.173
Triglycerides (mmol/L)	1.50 ± 1.11	1.34 ± 0.09	1.40 ± 0.12	−0.06 ± 0.08	0.449
HDL cholesterol (mmol/L)	1.36 ± 0.26	1.29 ± 0.04	1.30 ± 0.04	−0.07 ± 0.02	0.499
LDL cholesterol (mmol/L)	3.82 ± 0.93	3.78 ± 0.15	3.85 ± 0.16	−0.17 ± 0.10	0.504
Total cholesterol (mmol/L)	5.80 ± 0.98	5.70 ± 0.15	5.83 ± 0.16	−0.13 ± 0.09	0.163
Total/HDL ratio	4.39 ± 1.11	4.46 ± 0.20	4.63 ± 0.20	−0.07 ± 0.09	0.438

### 3.2. Safety and Tolerability

Throughout the 12-week intervention, there was only one serious adverse event (SAE), which involved hospitalisation of an individual who had been scheduled, prior to study enrolment, for a routine angiogram check, which required an overnight stay in the hospital. The purpose of the angiogram was to determine the appropriate size of an aortic stent to manage the participant’s aneurysm. The participant was readmitted to the hospital for the stent procedure and discharged without incident. The participant completed the intervention within the study timeframe. The SAE and subsequent follow-up was reported to the Human Ethics Research Committee of the University of Newcastle.

There were four reported adverse events during the 12-week intervention. The complaints were vivid dreams (*n* = 1), gastrointestinal discomfort (*n* = 1), increased headache frequency and severity for a pre-existing migraine sufferer (*n* = 1) and improved taste (*n* = 1). *Post hoc* analysis revealed that the first three complaints occurred during the active treatment phase.

### 3.3. Clinic Blood Pressure, Heart Rate and Arterial Compliance

Clinic SBP and DBP readings at the end of the initial treatment phase were lower than those taken at the screening/baseline visit (Table 1). However, there were no order effects of treatment, and compared with placebo, regular consumption of the combined formulation did not have an effect on clinic SBP, or DBP, or HR. Large artery elasticity tended to be lower at the end of the active treatment phase, but the difference was not significant following adjustment for multiple comparisons (Table 1). A parallel comparison was also made of the change from baseline to six weeks between those randomised initially to the combined formulation and those who received the placebo (see the table in the online Data Supplement). This comparison also failed to show any significance difference in the clinical measures of BP, HR and AC. Additionally, there was no gender difference in these measures.

### 3.4. Ambulatory Blood Pressure Monitoring

Compared with the placebo treatment, daily consumption of the combined formulation for six weeks did not affect 24-h, daytime nor nocturnal averages of ambulatory measures of SBP, DBP, MAP and HR (Table 2). Once again, there was no gender difference, and the analysis was not compromised by any order effect. A parallel comparison was also made of the change from baseline to six weeks between those randomised initially to the combined formulation and those who received the placebo (see the table in the online Data Supplement). This comparison also failed to show any significance difference in the ambulatory measures of SBP, DBP, MAP and HR. Additionally, there was no gender difference in these measures.

### 3.5. Blood Biochemistry

Fasting blood lipids, glucose levels and insulin sensitivity (HOMA-Index) were also unaffected by treatment (Table 3). Nor were any differences detected, once again, in a parallel comparison between active and placebo treatment arms in the first phase of the intervention. However, retrospective analysis revealed a gender difference in the crossover comparison. In men taking the combined formulation, there was a significant decrease in total cholesterol (active: 5.69 ± 0.21 mmol/L; placebo: 5.96 ± 0.21 mmol/L; change = −0.27 ± 0.09 mmol/L, *p* = 0.006; *n* = 20). This decrease in total cholesterol was accompanied by lowering of HDL cholesterol (active: 1.18 ± 0.04 mmol/L; placebo: 1.24 ± 0.05 mmol/L; change = −0.06 ± 0.0 mmol/L, *p* = 0.014) and LDL cholesterol (active: 3.86 ± 0.19 mmol/L; placebo: 4.03 ± 0.20 mmol/L; change = −0.18 ± 0.09 mmol/L, *p* = 0.055). This result was not compromised by order effects or differences in baseline characteristics between Group 1 and Group 2. In women, the blood biomarkers remained unchanged following treatment.

## 4. Discussion

This is the first study to investigate the effects of supplementation with this combined formulation for six weeks on 24-h ABP, clinic BP and AC, blood lipids, glucose and insulin sensitivity in adults with mildly-elevated, but untreated, hypertension. We found no significant effect of the combined formulation on any physiological nor biochemical measures of cardiometabolic risk in our study. The daily dose of olive leaf extract (1000 mg) accounted for more than 60% of the active ingredients in the combined formulation and contained 160–240 mg of oleuropein (calculated from Benolea^®^ EFLA^®^943 Product Flyer). Similarly, in the study by de Bock *et al*., overweight men who were supplemented daily with 380 mg of olive leaf extract (containing 51.1 mg of oleuropein) for 12 weeks did not show improvements in 24-h ABP or arterial elasticity, as determined by carotid intima-media thickness; however, these improvements would not be expected in a normotensive cohort [17]. The blood pressure lowering mechanisms of oleuropein are unclear, although *in vitro* evidence exits [18].

In published human studies on the effects of olive leaf extract on clinic BP [2,3] and biomarkers of diabetes [4,17], participants were supplemented daily for 8, 14 and 12 weeks, respectively. Susalit *et al*. [3] reported a small reduction in clinic SBP, triglycerides, total and LDL cholesterol compared to baseline values after eight weeks of olive leaf extract supplementation. While the dose of olive leaf extract used was the same as in the current study, the lack of a placebo comparison confounds the interpretation of their findings. Although they used a comparator drug, they doubled the dose of the comparator in patients who did not show a BP reduction at Week 2 of the eight-week intervention, thereby introducing a major methodological flaw in their evaluation. In another eight-week olive leaf extract supplementation study in borderline hypertensive monozygotic twins [2], Perrinjaquet-Moccetti *et al*. found a significant reduction in clinic BP (SBP, DBP and MAP) from baseline values at the sixth week of intervention, which returned to baseline after eight weeks. This reversion suggests that the antihypertensive effects may be transient and not sustainable. They also reported reductions in total and LDL cholesterol from baseline values following 1000 mg/day olive leaf extract consumption [2]. Both studies [2,3] were conducted by the supplement manufacturer. Once again, it is difficult to interpret the efficacy of olive leaf extract due to the lack of a placebo comparison. This introduces serious biases, particularly in relation to clinical assessment of BP, where the potential for a regression to the mean effect is well recognised [19]. This artefact was avoided in the present study by the use of ABP and a placebo control. Whilst there was a 10-mmHg reduction in clinic SBP from baseline to the end of the first six-week intervention phase, no such reduction occurred with ABP measurement. The lack of improvement in the 24-h, daytime and nocturnal ABP averages following active supplementation confirms the absence of BP lowering effects in the combined formulation in our present study. This was also evident from the clinic BP results in the present study, where a placebo comparison was used.

In the present study, we noted a gender difference in the lipid response; we observed a 5% reduction in total cholesterol following six weeks of consumption of the combined formulation; however, this resulted from reductions of both HDL and LDL cholesterol. Nonetheless, in high-risk individuals, a 10% reduction of serum total cholesterol is estimated to confer an 8%–10% reduction in coronary heart disease mortality risk [20]. Interestingly, de Bock *et al*. [21] found a marked gender difference in the absorption and metabolism of oleuropein; males were more efficient at conjugating oleuropein. De Bock *et al*. [17] also found a 15%–20% improvement in insulin sensitivity, compared with placebo, following 12 weeks of daily supplementation with 380 mg of olive leaf extract (containing 51.1 mg of oleuropein) in overweight men with borderline insulin resistance (mean Matsuda Index = 5.12; Matsuda Index <4.3 indicates insulin resistance). Similarly, daily olive leaf extract supplementation (500 mg/day for 14 weeks) lowered HbA1C and fasting plasma insulin levels in adults with type 2 diabetes mellitus [4]. We did not see any improvements in insulin sensitivity following six weeks of supplementation with the combined formulation in our non-diabetic/insulin resistant cohort (mean HOMA-IR = 1.40), suggesting that a longer period of supplementation is needed to benefit those with insulin resistance.

Even though olive leaf extract, at the dose given, may have been ineffective, the combined formulation also contained green coffee bean extract with chlorogenic acid, which has been shown to have antihypertensive potential. Ferulic acid, a metabolite of chlorogenic acid, can enhance NO bioavailability *in vitro* [22], leading to endothelium-dependent vasodilatation, without affecting endothelium-independent sodium nitroprusside-induced vasodilatation [7]. The dose of green coffee bean extract used in the current study (which delivered 93 mg/day of chlorogenic acid) has been shown to lower BP in a dose-response study by Kozuma *et al*. [6]. Watanabe *et al*. [23] also reported an antihypertensive effect in untreated mildly hypertensive adults after consuming 140 mg/day of chlorogenic acid in a green coffee bean extract. However, the participants also underwent lifestyle modifications in a bid to lower their BP during the 12-week intervention (*i.e.*, weight, dietary sodium and alcohol reduction and/or increased physical activity), which might have contributed to the BP reduction in the active treatment group. The lifestyle modifications were not standardised or monitored by the authors. Zhao *et al*. [7] cautioned that these two clinical trials were carried out in Far East Asia and suggested that ethnicity or differences in dietary customs may affect their reproducibility. In a recent acute intervention conducted in healthy adults in Australia (a predominantly Caucasian population) by Mubarak *et al*. [24], clinic SBP/DBP was lowered by 2.4/1.5 mmHg 60–90 min after consuming 400 mg of pure chlorogenic acid mixed in water (equivalent to drinking two cups of coffee) compared with placebo (water). However, this antihypertensive effect was not accompanied by an increase in endogenous NO or enhancement of flow-mediated dilatation (FMD) of the brachial artery, a reliable NO-mediated endothelium-dependent vasodilatory response, thereby contradicting the abovementioned preclinical evidence [7]. We did not see any BP lowering changes following regular consumption of the combined formulation, indicating a lack of antihypertensive efficacy of a 93-mg daily dose of chlorogenic acid in our Caucasian participants.

The dose of beetroot powder used in this study (300 mg/day) was considerably lower than the equivalent obtained from an efficacious dose of at least 100 g of beetroot juice in Hobbs *et al*. [9]. Plasma nitrate has a half-life of 6 h [25] and is excreted within 24 h [26]; hence, it was unlikely that the small amount of beetroot powder consumed in the combined formulation would have had an independent effect on BP, although it might have enhanced an anticipated effect of the other components at the doses administered.

Considering that previous trials of the individual components of our combined formulation have claimed benefits for BP, blood lipids or insulin sensitivity, the outcome of our study is disappointing. One might argue that any potential effect could have been masked by a carryover effect of treatment in the crossover design, although there is no evidence to suggest that the bioactives in this combined formulation have persistent effects following wash-out. We had a sufficient number of participants completing the study to perform a parallel comparison of each treatment arm in the first six-week intervention phase, which confirmed a lack of effect on all outcome measures. Whilst unlikely, it is also possible that consuming a combination of three bioactive ingredients in a single tablet could have negatively impacted on the bioavailability of each individual ingredient or resulted in competing mechanistic actions on arterial function. This is a limitation that should be addressed by undertaking initial dose-response evaluations of the ingredients individually and using the results as a basis for formulating efficacious combinations.

## 5. Conclusions

In conclusion, six weeks of daily supplementation with a combined formulation, comprising extracts of olive leaf, green coffee bean and beetroot, did not lower 24-h ABP nor clinic BP nor improve blood lipids, blood glucose nor insulin sensitivity in adults with borderline or mildly-elevated BP. However, evidence of efficacy from previous trials of olive leaf extract is questionable. Further evaluation of the individual ingredients is required in well-designed, randomised, double-blind, placebo-controlled trials in target populations. The choice of ethnicity should also be a consideration for future study designs.

## Supplementary Files

Supplementary File 1
