# Supplementary material for: Antihypertensive Potential of Combined Extracts of Olive Leaf, Green Coffee Bean and Beetroot: A Randomized, Double-Blind, Placebo-Controlled Crossover Trial
Source: Nutrients. 2014 Nov 5;6(11):4881–94. doi: 10.3390/nu6114881 (PMC4245569; doi:10.3390/nu6114881)
Supplement: Supplementary File 1 [file nutrients-06-04881-s001.docx]

**Supplementary Information**

**Results**

Tables 1–3 below detail the parallel comparisons of the change in clinic BP, HR, AC, 24-h ABP, day-time BP, nocturnal BP averages and blood biochemistry of total, HDL, LDL cholesterol, glucose and insulin-sensitivity from baseline (Week 0) to six weeks between those randomised initially to the combined formulation and those who received the placebo.

**Table 1.** Parallel comparison of body mass index, clinic blood pressure, heart rate and arterial compliance at the end of the first intervention phase between the baseline change from active (combined formation) and from placebo. Data ± SEM.

| **Clinic BP, HR and arterial compliance** | **Change**  **(baseline-active)**  **(*n* = 19)** | **Change**  **(baseline-placebo)**  **(*n* = 18)** | ***p*** |
| --- | --- | --- | --- |
| BMI (kg/m^2^) | 0.05 ± 0.07 | −0.14 ± 0.11 | 0.136 |
| Clinic SBP (mmHg) | 9.56 ± 1.99 | 8.57 ± 2.44 | 0.755 |
| Clinic DBP (mmHg) | 4.37 ± 0.72 | 3.02 ± 1.05 | 0.292 |
| Clinic HR (bpm) | 2.81 ± 1.10 | 3.59 ± 1.39 | 0.658 |
| Clinic large artery elasticity index (mL/mmHg × 10) | −3.88 ± 1.05 | −2.21 ± 0.80 | 0.218 |
| Clinic small artery elasticity index (mL/mmHg × 100) | −0.65 ± 0.46 | 0.07 ± 0.27 | 0.198 |

**Table 2.** Parallel comparison of 24-h, day-time and nocturnal ABP averages at the end of the first intervention phase between the baseline change from active (combined formulation) and from placebo. Data ± SEM.

| **Ambulatory BP** | **Change**  **(baseline-active)**  **(*n* = 19)** | **Change**  **(baseline-placebo)**  **(*n* = 18)** | ***p*** |
| --- | --- | --- | --- |
| **24-h ambulatory BP average** | | | |
| SBP (mmHg) | 1.68 ± 1.62 | 2.22 ± 1.42 | 0.805 |
| DBP (mmHg) | 0.68 ± 0.80 | 1.17 ± 0.83 | 0.677 |
| MAP (mmHg) | 1.00 ± 1.04 | 1.56 ± 0.95 | 0.697 |
| HR (bpm) | 0.26 ± 0.60 | 1.00 ± 0.86 | 0.483 |
| **Day-time BP average (07:00–22:00)** | | | |
| SBP (mmHg) | 1.63 ± 1.54 | 3.00 ± 1.56 | 0.536 |
| DBP (mmHg) | 0.26 ± 0.90 | 1.50 ± 0.98 | 0.357 |
| MAP (mmHg) | 0.58 ± 0.97 | 2.11 ± 1.07 | 0.296 |
| HR (bpm) | 0.21 ± 0.74 | 1.11 ± 0.96 | 0.458 |
| **Nocturnal BP average (22:00-07:00)** | | | |
| SBP (mmHg) | 1.58 ± 2.69 | −0.15 ± 2.11 | 0.620 |
| DBP (mmHg) | 1.53 ± 1.33 | −0.22 ± 0.97 | 0.298 |
| MAP (mmHg) | 1.42 ± 1.68 | −0.28 ± 1.30 | 0.434 |
| HR (bpm) | 0.32 ± 0.95 | 0.72 ± 1.32 | 0.802 |

**Table 3.** Parallel comparison of blood biochemistry of total, HDL and LDL cholesterol, blood glucose and insulin levels and HOMA-index of insulin resistance at the end of the first intervention phase between the baseline change from active (combined formulation) and from placebo.

| **Biochemistry** | **Change**  **(baseline-active)**  **(*n* = 19)** | **Change**  **(baseline-placebo)**  **(*n* = 18)** | ***p*** |
| --- | --- | --- | --- |
| Glucose (mmol/L) | −0.08 ± 0.08 | −0.09 ± 0.08 | 0.894 |
| Insulin (mmol/L) | −0.64 ± 0.67 | −1.72 ± 0.93 | 0.346 |
| HOMA-IR | −0.17 ± 0.17 | −0.57 ± 0.22 | 0.148 |
| Triglycerides (mmol/L) | 0.37 ± 0.24 | −0.16 ± 0.13 | 0.063 |
| HDL cholesterol (mmol/L) | 0.05 ± 0.02 | 0.02 ± 0.03 | 0.526 |
| LDL cholesterol (mmol/L) | 0.11 ± 0.07 | 0.11 ± 0.15 | 0.964 |
| Total cholesterol (mmol/L) | 0.19 ± 0.07 | −0.06 ± 0.14 | 0.108 |
| Total/HDL Ratio | 0.02 ± 0.08 | −0.08 ± 0.10 | 0.425 |

© 2014 by the authors; licensee MDPI, Basel, Switzerland. This article is an open access article distributed under the terms and conditions of the Creative Commons Attribution license (http://creativecommons.org/licenses/by/4.0/).
